# Access to Original Vinylic Chlorides in the Quinazoline Series via a Monoelectronic Transfer Reaction Approach

**DOI:** 10.3390/molecules15042719

**Published:** 2010-04-13

**Authors:** Martine Maillard-Boyer, Caroline Castera-Ducros, Pierre Verhaeghe, France Sifredi, Pascal Rathelot, Patrice Vanelle

**Affiliations:** Laboratoire de Pharmacochimie Radicalaire, Faculté de Pharmacie, Universités d’Aix-Marseille I, II et III - UMR CNRS 6264, Laboratoire Chimie Provence, 27 Boulevard Jean Moulin, 13385 Marseille cedex 05, France; E-Mails: martine.maillard@univmed.fr (M.M.); caroline.ducros@univmed.fr (C.C.); pierre.verhaeghe@univmed.fr (P.V.); france.sifredi@univmed.fr (F.S.); pascal.rathelot@univmed.fr (P.R.)

**Keywords:** S_RN_1, E_RC_1, quinazoline ring, vinylic chlorides

## Abstract

A series of new quinazoline derivatives bearing a vinylic chloride group on the 2-position was prepared by using a consecutive S_RN_1 / E_RC_1 radical strategy.

## 1. Introduction

The quinazoline ring is of major pharmaceutical interest, considering both that it is the molecular scaffold of several drug-compounds (Erlotinib, Lapatinib, Prazozin, Alfuzosin…) and, that 4-anilino-substituted-quinazolines are key structures for the development of new selective anticancer chemotherapies, as kinase inhibitors. Focusing on the synthesis of new 2,4-bisubstituted-quinazolines, with a scope of studying their biological properties, our research team also discovered that some 4-anilinoquinazolines have original *in vitro* antiplasmodial properties [1,2]. Quite recently, we prepared some 4-anilinoquinazolines, substituted at position 6 with a nitro group and at position 2 with a vinylic group, one of them exerting *in vitro* antiplasmodial properties [3]. To explore more precisely the relations between chemical structure and biological activity, we studied the possibility of synthesizing new 4-aminated-quinazoline derivatives presenting a vinylic chloride substituent at position 2. In order to access to such vinylic chloride group, different strategies were developed among which the one presented by Ryabukhin *et al.* [4] and the use of trichloromethylated heterocycles, as substrates for the radical S_RN_1+E_RC_1 reaction with nitroalkanes, successful approach in isoquinoline, imidazole and quinoline series [5,6,7]. We describe herein, the use of the consecutive S_RN_1+E_RC_1 reaction from 2-trichloromethylquinazoline substrates and nitroalkanes, for the preparation of these original vinylic chloride derivatives in a rapid and convenient way.

## 2. Results and Discussion

Since 2009, it is known that 2-chloromethyl-6-nitroquinazoline derivatives can react with nitroalkanes through a S_RN_1 mechanism [8]. However, the S_RN_1 reaction involving a radical anion intermediate which is generally stabilized with the presence of a nitro group properly located on the heterocycle, it was not obvious that an aminated quinazoline substrate would react, because of the electrodonating effect of this group.

**Scheme 1:**
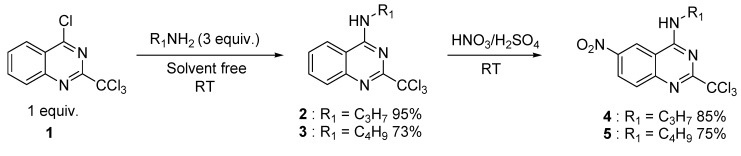
Two step preparation of the radical reaction substrates **4** and **5**.

First, the key-precursor **1** was prepared from commercial 2-methylquinazolin-4(3*H*)-one *via* a microwave-assisted chlorination procedure using phosphorus pentachloride in phosphorus oxychloride [9]. Then, after proceeding to a solvent free S_N_Ar reaction with *n*-propyl- and *n*-butylamines, the corresponding intermediates **2** and **3** were nitrated in classical conditions to afford **4** and **5** in good yields (Scheme 1).

**Scheme 2:**
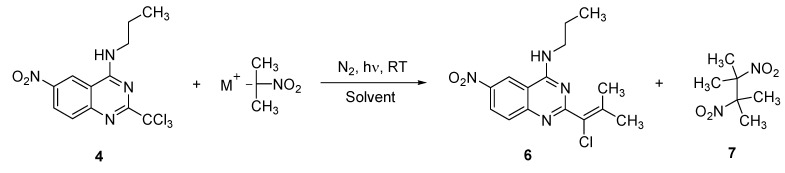
General presentation of the S_RN_1+E_RC_1 reaction of substrate **4** with 2-nitropropane.

Then, compound **4**, which is prepared in higher global yield, was chosen as a substrate for studying the SRN1+ERC1 reaction with 2-nitropropane. The first reaction assay, at room temperature (RT) under N2 atmosphere and light irradiation, enabled us to isolate both vinylic chloride **6** and the dimer of 2-nitropropane **7** (Scheme 2).

In order to optimize the reaction, several experimental conditions were studied, especially the ones described by Kornblum *et al*. [10] (lithium salt of 2-nitropropane in DMSO or DMF) and Norris *et al.* [11] (phase transfer conditions using TBAOH and 2-nitropropane). The results showed that the phase transfer conditions of Norris gave the best reaction yield. The amount of 2-nitropropane required was also studied, indicating that the reaction yield is globally optimal (82%) when using 5 equiv. (Table 1, entries 1–6). Reaction progress was monitored by TLC, and appeared to be complete after 4 hours.

The formation of dimer **7** clearly suggested a consecutive S_RN_1+E_RC_1 reaction mechanism. To validate this hypothesis, we studied the influence of several radical reaction inhibitors (Table 1 entries 7–10). The results showed that the reaction obeys to a radical pathway, in particular when comparing run 3 with runs 8 or 9. The synthesis of vinylic chloride **6** in low yield, when using 1 equiv. of *p*-dinitrobenzene (assay 10), may be due to the insufficient difference in reduction potential between *p*-dinitrobenzene and substrate **4**, partially permitting the monoelectronic reduction of this last by the 2-nitropropane anion, followed by the formation of product **6**, through complete S_RN_1+E_RC_1 reaction sequence.

**Table 1:** Reactivity of substrate **4** with 2-nitropropane.

Run*	M^+^	Solvent	2-Nitropropane (equiv.)	Inhibitor	Yield of 6 (%)
**1**	NBu_4_	CH_2_Cl_2_H_2_O	2	-	60
**2**	NBu_4_	CH_2_Cl_2_H_2_O	4	-	75
**3**	NBu_4_	CH_2_Cl_2_H_2_O	5	-	82
**4**	NBu_4_	CH_2_Cl_2_H_2_O	6	-	83
**5**	Li	DMF	5	-	53
**6**	Li	DMSO	5	-	70
**7**	Li	DMSO	5	CuCl_2_ (1 equiv.)	25
**8**	NBu_4_	CH_2_Cl_2_H_2_O	5	TEMPO (1 equiv.)	0
**9**	NBu_4_	CH_2_Cl_2_H_2_O	5	O_2_ bubbling	0
**10**	NBu_4_	CH_2_Cl_2_H_2_O	5	*p*-dinitrobenzene	35

*Reaction conditions: N_2_ atmosphere, 60W white light irradiation, RT. Reactions were monitored by TLC.

**Scheme 3:**
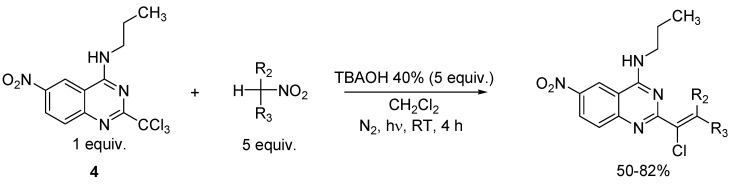
Optimal reaction conditions for the synthesis of vinylic chloride derivatives from **4**.

From the optimal reaction conditions previously presented, we then extended the reaction to other nitroalkanes, in order to prepare several vinylic chlorides in quinazoline series (Scheme 3). So the same radical reaction was conducted with two other secondary nitroalkanes (nitrocycloalkanes) and three primary nitroalkanes. In the reactions using secondary nitroalkanes, the corresponding vinylic chlorides were obtained in identical yields (60–82%) as with nitroalkane dimers, whereas in the reactions involving primary nitroalkanes, only the corresponding vinylic chlorides were obtained, in significantly lower yields (50–56%), as presented in Table 2. Concerning the vinylic chlorides prepared from primary nitroalkanes, the analysis of 2D Noesy NMR spectra revealed a *Z* configuration for the vinylic double bond.

Such results appeared identical to the ones obtained with the S_RN_1+E_RC_1 reaction operated in 2-trichloromethylquinoline series [7], but demonstrate that this reaction can be compatible with the presence of an electrodonating group on the heterocyclic scaffold.

**Table 2:** Series of vinylic chloride derivatives prepared from **4**, using optimal reaction conditions.

Nitroalkane	Vinylic chloride	Dimer	**Yield of vinylic chloride (%)**
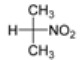	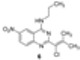	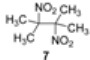	**82**
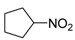	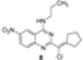	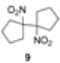	**60**
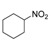	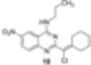	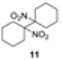	**66**
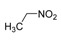	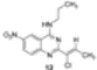	Not isolated	**56**
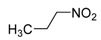	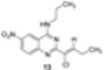	Not isolated	**50**
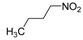	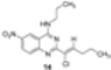	Not isolated	**52**

**Scheme 4:**
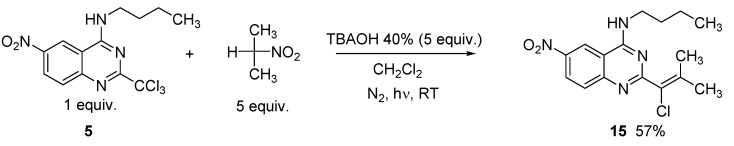
Extension of the reaction with substrate **5**.

To enlarge the reaction spectrum, we involved two other aminated substrates in the same reaction with 2-nitropropane, using the previously optimal reaction conditions. Substrate **5**, containing an other primary amine substituent, was prepared in parallel with **4** and afforded vinylic chloride **15** in 57 % yield (Scheme 4).

**Scheme 5:**
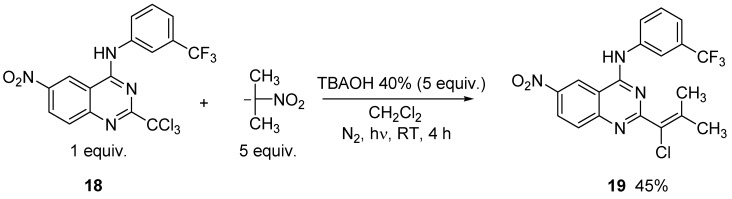
Three step preparation of substrate **18**.

Aiming at preparing a vinylic chloride product bearing an anilino substituent at position 4 of quinazoline, we had to prepare an appropriate substrate candidate for the S_RN_1+E_RC_1 reaction. Thus, since the nitration reaction could not be done after the introduction of the aniline onto the quinazoline ring, the synthetic strategy from 2-methylquinazolin-4(3*H*)-one was modified, starting by a nitration step, under classical conditions. After successive chlorination and solvent free S_N_Ar reactions, **18** was obtained (Scheme 5).

**Scheme 6:**
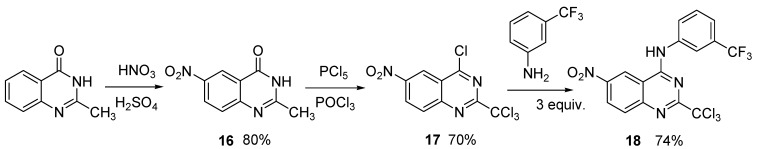
S_RN_1+E_RC_1 reaction of **18** with 2-nitropropane.

So we applied the optimal reaction conditions defined previously to the reaction of **18** with 2-nitropropane and obtained the expected vinylic chloride **19** in 45% yield (Scheme 6).

**Scheme 7:**
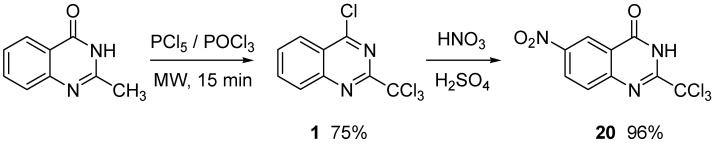
Two step preparation of substrate **20**.

When 2-methylquinazolin-4(3*H*)-one is first chlorinated and then nitrated, compound 20 is formed in 72% global yield (Scheme 7).

**Scheme 8:**
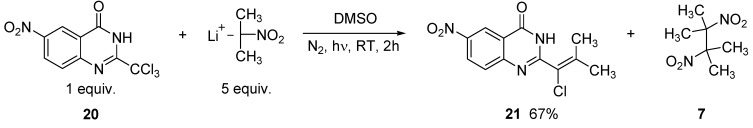
S_RN_1+E_RC_1 reaction of **20** with the lithium salt of 2-nitropropane.

Finally, because of its trichloromethyl group, we thought that such nitrated lactam ring 20 could be an interesting substrate for the S_RN_1+E_RC_1 reaction. Unfortunately, its low solubility in dichloromethane did not allow the use of the optimal phase transfer reaction conditions that we already presented. So we reacted **20** with the lithium salt of 2-nitropropane in DMSO (Kornblum conditions [10]) during 2 hours and formed the vinylic chloride product **21** in 67 % yield, in addition to the dimer of 2-nitropropane **7**, in similar yield (Scheme 8).

## 3. Experimental

### 3.1. General

Melting points were determined with a B-540 Büchi melting point apparatus. 200 MHz ^1^H-NMR and 50 MHz ^13^C-NMR spectra were recorded on a Brüker ARX 200 spectrometer in CDCl_3_ or DMSO-d_6_ at the Faculté de Pharmacie de Marseille. ^1^H- and ^13^C-NMR chemical shifts (δ) are reported in ppm with respect to CDCl_3_ 7.26 ppm (^1^H), 77.2 ppm (^13^C) or DMSO-d_6_ 2.50 ppm (^1^H), 39.5 (^13^C). 2D NOESY NMR spectra and elemental analyses were carried out at the Spectropole, Faculté des Sciences et Techniques de Saint-Jérôme. Silica gel 60 (Merck, particular size 0.063–0.200 mm, 70–230 mesh ASTM) was used for column chromatography. TLC was performed on 5 cm × 10 cm aluminium plates coated with silica gel 60F-254 (Merck) in appropriate solvent. The following substrates or subproducts were prepared and described previously: 4-chloro-2-trichloromethyl-quinazoline (**1**) [9,12], *N*-butyl-2-trichloromethylquinazolin-4-amine (**3**) [13], 2,3-dimethyl-2,3-dinitrobutane (**7**) [14], 1-nitro-1-(1-nitrocyclopentyl)cyclopentane (**9**) [15], 1-nitro-1-(1-nitrocyclo-hexyl)cyclohexane (**11**) [16], 2-methyl-6-nitroquinazolin-4(3*H*)one (**16**) [17], 4-chloro-6-nitro-2-trichloromethylquinazoline (**17**) [18], and 6-nitro-2-trichloromethylquinazolin-4(*3H*)-one (**20**) [18].

### 3.2. Preparation of N-propyl-2-trichloromethylquinazolin-4-amine *(**2**)*

Three equiv. of propylamine were added onto 1 equiv. of 4-chloro-2-trichloromethylquinazoline (**1**). The solvent-free reaction mixture was stirred at 30 °C for 1 min. The mixture was poured into a solution of NaOH (5%) and extracted with dichloromethane. The organic layer was washed with water three times, dried over anhydrous Na_2_SO_4_, concentrated in vacuo and recrystalized from cyclohexane to afford **2** in 95% yield.Beige solid; yield 95%, mp 136 °C. ^1^H-NMR (CDCl_3_) *δ* 1.03 (3H, t, *J* = 7.3 Hz), 1.69–1.87 (2H, m), 3.66–3.76 (2H, m), 6.06 (1H, br s), 7.49–7.57 (1H, m), 7.73–7.81 (2H, m), 7.97 (1H, d, *J* = 8.3 Hz). ^13^C-NMR (50 MHz; CDCl_3_) *δ* 11.6 (CH_3_), 22.5 (CH_2_), 43.4 (CH_2_), 98.4 (C), 113.8 (C), 121.2 (CH), 127.4 (CH), 129.0 (CH), 133.2 (CH), 149.1 (C), 160.7 (C), 161.5 (C). Anal. calcd for C_12_H_12_Cl_3_N_3_: C, 47.32; H, 3.97; N, 13.80. Found: C, 47.90; H, 4.24; N, 13.85.

### 3.3. General Procedure for the Preparation of Compounds ***4*** and ***5***

To concentrated sulfuric acid (25 mL), 1 equiv. of *N*-alkyl-2-trichloromethylquinazolin-4-amine (**2**) or **3** was added at 0 °C. Then, 5 equiv. of concentrated nitric acid were added slowly. The reaction mixture was stirred for 5 h at rt. The reaction mixture was then slowly poured into a water-ice mixture and alkalinized with sodium carbonate, making the desired products precipitate. The suspension was filtered and the precipitate was dried under reduced pressure. After purification by chromatography on a silica gel column, eluting with dichloromethane, products **4** or **5** were recrystallized from ethyl acetate and obtained, respectively, in 85 and 75% yields.

*6-Nitro-N-propyl-2-trichloromethylquinazolin-4-amine* (**4**): Yellow solid; yield 85%, mp 151 °C. ^1^H-NMR (CDCl_3_) *δ* 1.07 (3H, t, *J* = 7.3 Hz), 1.76-1.94 (2H, m), 3.74–3.84 (2H, m), 6.58 (1H, br s), 8.07 (1H, d, *J* = 9.1 Hz), 8.54 (1H, dd, *J* = 9.1 Hz and *J* = 2.4 Hz), 8.83 (1H, d, *J* = 2.4 Hz). ^13^C-NMR (CDCl_3_) *δ* 11.7 (CH_3_), 22,5 (CH_2_), 44.2 (CH_2_), 97.3 (C), 113.1 (C), 118.5 (CH), 127,2 (CH), 130.8 (CH), 145.7 (C), 152.7 (C), 161.6 (C), 164.4 (C). Anal. Calcd for C_12_H_11_Cl_3_N_4_O_2_: C, 41.23; H, 3.17; N, 16.03. Found: C, 41.74; H, 3.05; N, 16.45.

*N-Butyl-6-nitro-2-trichloromethylquinazolin-4-amine* (**5**): Yellow solid; yield 75%, mp 168 °C. ^1^H-NMR (CDCl_3_) *δ* 1.01 (3H, t, *J* = 7.3 Hz), 1.40–1.59 (2H, m), 1.75–1.87 (2H, m), 3.82 (2H, q, *J* = 12.7 Hz), 6.75 (1H, br s), 8.07 (1H, d, *J* = 9.2 Hz), 8.55 (1H, dd, *J* = 9.2 and 2.3 Hz), 8.83 (1H, d, *J* = 2.3 Hz). ^13^C-NMR (CDCl_3_) *δ* 13.8 (CH_3_), 20.1 (CH_2_), 31.1 (CH_2_), 41.9 (CH_2_), 97.3 (C), 112.9 (C), 118.0 (CH), 126.9 (CH), 131.1 (CH), 145.5 (C), 153.0 (C), 161.3 (C), 164.5 (C). Anal. Calcd for C_13_H_13_Cl_3_N_4_O_2_: C, 42.94; H, 3.60; N, 15.41. Found: C, 43.01; H, 3.63; N, 15.25.

### 3.4. General Procedure for the Synthesis of Vinylic Chlorides ***6, 8, 10, 12, 13, 14, 15*** and ***19***

Five equiv. each of both nitroalkane and 40% TBAOH were stirred at room temperature for 30 min under nitrogen atmosphere. Then, 1 equiv. of 4-substituted-6-nitro-2-trichloromethylquinazoline substrate **4**, **5** or **18**, solubilized in dichloromethane, was added. The mixture was rapidly stirred at rt under nitrogen atmosphere with white light irradiation (60 W) for 4 h. After disappearance of substrate (monitored by TLC), the reaction was stopped with addition of water and the product was extracted with dichloromethane. The organic layer, washed with water several times, was finally dried over anhydrous sodium sulphate and evaporated. The product was purified by chromatography on a silica gel, eluting with appropriate solvent, to give the corresponding vinylic chloride products.

*2-(1-Chloro-2-methylprop-1-enyl)-6-nitro-N-propylquinazolin-4-amine* (**6**): The eluent was petroleum ether-ethyl acetate 50:50. Yellow solid; yield 82%, mp 145 °C. ^1^H-NMR (CDCl_3_) *δ* 1.06 (3H, t, *J* = 7.3 Hz), 1.73–1.86 (2H, m), 2.05 (3H, s), 2.09 (3H, s), 3.66–3.76 (2H, m), 6.29 (1H, br s), 7.94 (1H, d, *J* = 9.2 Hz), 8.48 (1H, dd, *J* = 9.2 Hz and 2.4 Hz), 8.76 (1H, d, *J* = 2.4 Hz). ^13^C-NMR (CDCl_3_) *δ* 11.7 (CH_3_), 22.4 (CH_3_), 22.7 (CH_2_), 23.3 (CH_3_), 43.7 (CH_2_), 112.5 (C), 118.4 (CH), 124.5 (C), 126.6 (CH), 130.1 (CH), 137.7 (C), 144.7 (C), 153.4 (C), 160.7 (C), 164.2 (C). Anal. Calcd for C_15_H_17_ClN_4_O_2_: C, 56.16; H, 5.34; N, 17.47. Found: C, 56.75; H, 5.69; N, 17.05.

*2-[Chloro(cyclopentylidene)methyl]-6-nitro-N-propylquinazolin-4-amine* (**8**): The eluent was dichloromethane-ethyl acetate 98:2. Orange solid; yield 60%, mp 160 °C. ^1^H-NMR (DMSO-d_6_) *δ* 0.95 (3H, t, *J* = 7.3 Hz), 1.59–1.80 (6H, m), 2.59–2.65 (2H, m), 2.92–2.98 (2H, m), 3.49–3.51 (2H, m), 7.76 (1H, d, *J* = 9.2 Hz), 8.42 (1H, dd, *J* = 9.2 Hz and 2.3 Hz), 8.98–9.00 (1H, m), 9.31 (1H, d, *J* = 2.3 Hz). ^13^C-NMR (DMSO-d_6_) *δ* 11.7 (CH_3_), 21.8 (CH_2_), 25.1 (CH_2_), 27.8 (CH_2_), 35.2 (CH_2_), 36.6 (CH_2_), 43.0 (CH_2_), 112.3 (C), 120.6 (CH), 122.4 (C), 126.5 (CH), 129.2 (CH), 144.0 (C), 153.4 (C), 154.2 (C), 160.2 (C), 161.9 (C). HR MS (+ESI): *m/z* 347.1269 (M+H^+^). Calcd for C_17_H_19_ClN_4_O_2_: 346.1197.

*2-[Chloro(cyclohexylidene)methyl]-6-nitro-N-propylquinazolin-4-amine* (**10**): The eluent was petroleum ether-ethyl acetate 50:50. Yellow solid; yield 66%, mp 170 °C. ^1^H-NMR (CDCl_3_) *δ* 1.05 (3H, t, *J* = 7.3 Hz), 1.61–1.85 (10H, m), 2.57–2.62 (2H, m), 3.66–3.76 (2H, m), 6.37 (1H, br s), 7.93 (1H, d, *J* = 9.2 Hz), 8.46 (1H, dd, *J* = 9.2 Hz and 2.4 Hz), 8.77 (1H, d, *J* = 2.4 Hz). ^13^C-NMR (CDCl_3_) *δ* 11.7 (CH_3_), 22.7 (CH_2_), 26.4 (CH_2_), 27.3 (CH_2_), 27.9 (CH_2_), 30.0 (CH_2_), 32.4 (CH_2_), 43.7 (CH_2_), 112.4 (C), 118.4 (CH), 121.5 (C), 126.6 (CH), 130.2 (CH), 143.8 (C), 144.7 (C), 153.6 (C), 160.8 (C), 164.3 (C). HR MS (+ESI): *m/z* 361.1426 (M+H^+^). Calcd for C_18_H_21_ClN_4_O_2_: 360.1353.

*(Z)-2-(1-Chloroprop-1-enyl)-6-nitro-N-propylquinazolin-4-amine* (**12**): The eluent was dichloro-methane. Green solid; yield 56%, mp 176 °C. ^1^H-NMR (CDCl_3_) *δ* 1.03–1.10 (3H, m), 1.72–1.90 (2H, m), 2.08 (3H, d, *J* = 7.0 Hz ), 3.67–3.77 (2H, m), 6.43 (1H, br s), 7.63 (1H, q, *J* = 7.0 Hz), 7.92–7.98 (1H, m), 8.44 (1H, dd, *J* = 9.2 Hz and 2.4 Hz)), 8.78 (1H, d, *J* = 2.4 Hz). ^13^C-NMR (CDCl_3_) *δ* 11.8 (CH_3_), 15.8 (CH_3_), 22.5 (CH_2_), 43.9 (CH_2_), 112.9 (C), 118.9 (CH), 126.9 (CH), 129.4 (CH), 132.4 (C), 134.5 (CH), 144.6 (C), 152.5 (C), 160.0 (C), 160.5 (C). HR MS (+ESI): *m/z* 307.0956 (M+H^+^). Calcd for C_14_H_15_ClN_4_O_2_: 306.0884.

*(Z)-2-(1-Chlorobut-1-enyl)-6-nitro-N-propylquinazolin-4-amine* (**13**): The eluent was dichloro-methane. Yellow solid; yield 50%, mp 163 °C. ^1^H-NMR (CDCl_3_) *δ* 0.98–1.23 (6H, m), 1.77–1.88 (2H, m), 2.46–2.61 (2H, m), 3.68–3.78 (2H, m), 6.40 (1H, br s), 7.55 (1H, t, *J* = 9.3 Hz), 7.97 (1H, d, *J* = 9.2 Hz), 8.45 (1H, dd, *J* = 9.2 Hz and 2.4 Hz), 8.78 (1H, d, *J* = 2.4 Hz). ^13^C-NMR (CDCl_3_) *δ* 11.2 (CH_3_), 12.2 (CH_3_), 22.0 (CH_2_), 22.9 (CH_2_), 43.1 (CH_2_), 112.3 (C), 118.0 (CH), 126.1 (CH), 129.3 (CH), 130.8 (C), 139.8 (CH), 143.8 (C), 152.6 (C), 159.4 (C), 160.3 (C). Anal. Calcd for C_15_H_17_ClN_4_O_2_: C, 56.16; H, 5.34; N, 17.47. Found: C, 55.82; H, 5.75; N, 17.01.

*(Z)-2-(1-Chloropent-1-enyl)-6-nitro-N-propylquinazolin-4-amine* (**14**): The eluent was dichloromethane-ethyl acetate 99:1. Yellow solid; yield 52%, mp 156 °C. ^1^H-NMR (DMSO-d_6_) *δ* 0.92–0.99 (6H, m), 1.49–1.76 (4H, m), 2.36–2.44 (2H, m), 3.50–3.60 (2H, m), 7.46–7.53 (1H, m), 7.81 (1H, d, *J* = 9.1 Hz), 8.43 (1H, dd, *J* = 9.1 Hz and 2.0 Hz), 9.01 (1H, br s), 9.33 (1H, d, *J* = 2.0 Hz). ^13^C-NMR (DMSO-d_6_) *δ* 12.1 (CH_3_), 14.2 (CH_3_), 21.6 (CH_2_), 21.9 (CH_2_), 31.7 (CH_2_), 43.2 (CH_2_), 113.5 (C), 120.9 (CH), 126.9 (CH), 129.7 (CH), 132.6 (C), 137.1 (CH), 144.3 (C), 153.8 (C), 160.2 (C), 160.4 (C). HR MS (+ESI): *m/z* 335.1269 (M+H^+^). Calcd for C_16_H_19_ClN_4_O_2_: 334.1197.

*N-Butyl-2-(1-chloro-2-methylprop-1-enyl)-6-nitroquinazolin-4-amine* (**15**): The eluent was petroleum ether-ethyl acetate 50:50. Beige solid; yield 57%, mp 135 °C. ^1^H-NMR (CDCl_3_) *δ* 1.00 (3H, t, *J* = 7.2 Hz), 1.40–1.58 (2H, m), 1.72–1.84 (2H, m), 2.05 (3H, s), 2.09 (3H, s), 3.75 (2H, m), 6.42 (1H, br s), 7.96 (1H, d, *J* = 9.2 Hz), 8.49 (1H, dd, *J* = 9.2 and 2.3 Hz), 8.73 (1H, d, *J* = 2.3 Hz). ^13^C-NMR (CDCl_3_)*δ* 13.8 (CH_3_), 20.2 (CH_2_), 22.2 (CH_3_), 23.1 (CH_3_), 31.3 (CH_2_), 41.6 (CH_2_), 112.3 (C), 118.2 (CH), 124.4 (C), 126.4 (CH), 130.0 (CH), 137.4 (C), 144.5 (C), 153.3 (C), 160.5 (C), 164.0 (C). Anal. Calcd for C_16_H_19_ClN_4_O_2_: C, 57.40; H, 5.72; N, 16.73. Found: C, 57.02; H, 5.94; N, 16.43.

*2-(1-Chloro-2-methylprop-1-enyl)-6-nitro-N-[3-(trifluoromethyl)]-phenylquinazolin-4-amine* (**19**): The eluent was dichloromethane. Yellow solid, yield 45%, mp 201 °C. ^1^H-NMR (CDCl_3_) *δ* 2.13 (6H, s), 7.45–7.60 (2H, m), 7.98-8.11 (3H, m), 8.40 (1H, br s), 8.58 (1H, dd, *J* = 9.1 Hz and 2.2 Hz), 9.07 (1H, d, *J* = 1.7 Hz). ^13^C-NMR (CDCl_3_) *δ* 22.5 (CH_3_), 23.7 (CH_3_), 112.3 (C), 118.0 (CH), 118.8 (CH, q, *J* = 4.0 Hz), 121.6 (CH, q, *J* = 4.0 Hz), 123.8 (C, q, *J* = 272.6 Hz), 124.1 (C), 124.7 (CH), 127.0 (CH), 129.7 (CH), 130.6 (CH), 131.6 (C, q, *J* = 32.6 Hz), 138.0 (C), 140.1 (C), 145.2 (C), 153.3 (C), 158.2 (C), 163.1 (C). Anal. calcd for C_19_H_14_ClF_3_N_4_O_2_: C, 53.98; H, 3.34; N, 13.25. Found: C, 54.4; H, 3.52; N, 12.95.

### 3.5. Preparation of 6-nitro-2-trichloromethyl-N-[(3-trifluoromethyl)]phenylquinazolin-4-amine *(**18**)*

Three equiv. of 3-trifluoromethylaniline were added onto 1 equiv. (500 mg) of 4-chloro-6-nitro-2-trichloromethylquinazoline (**17**). The solvent-free reaction mixture was stirred at 120 °C for 15 min. The mixture was poured into a saturated solution of sodium carbonate and extracted with dichloromethane. The organic layer was washed with water three times, dried over anhydrous Na_2_SO_4_ and concentrated *in vacuo*. After purification by chromatography (silica gel, eluent: dichloromethane) the product was obtained in 74% yield. Yellow solid; yield 74%, mp 234 °C. ^1^H-NMR (DMSO-d_6_) *δ* 7.56 (1H, d, *J* = 7.7 Hz), 7.67–7.75 (1H, m), 8.14 (1H, d, *J* = 9.2 Hz), 8.22 (1H, d, *J* = 8.3 Hz), 8.65–8.71 (2H, m), 9.80 (1H, d, *J* = 2.4 Hz), 11.05 (1H, br s). ^13^C-NMR (DMSO-d_6_) *δ* 97.2 (C), 113.7 (C), 118.7 (CH, q, *J* = 4.0 Hz), 120.8 (CH), 120.9 (CH), 124.1 (CF_3_, q, *J* = 272.7 Hz), 125.5 (CH), 127.8 (CH), 129.3 (C, q, *J* = 31.9 Hz), 129.8 (CH), 130.4 (CH), 138.9 (C), 146.0 (C), 152.5 (C), 159.5 (C), 162.7 (C). Anal. calcd for C_16_H_8_Cl_3_F_3_N_4_O_2_: C, 42.55; H, 1.79; N, 12.41. Found: C, 42.50; H, 1.91; N, 11.97.

### 3.6. Preparation of 2-(1-chloro-2-methylprop-1-enyl)-6-nitroquinazolin-4(3H)-one *(**21**)*

To a DMSO solution (15 mL) of the lithium salt of 2-nitropropane (0.76 g, 8.1 mmol, 5 equiv.), a DMSO solution (15 mL) containing 0.5 g (1.6 mmol, 1 equiv.) of **20** was added rapidly. The mixture was stirred at rt for 2 h under N_2_ atmosphere and light irradiation (60W). After the disappearance of **20** (monitored by TLC), the mixture was poured into cold water, filtered, and extracted with dichloromethane. The combined organic layer were dried over anhydrous Na_2_SO_4_ and evaporated. The solid product was washed with petroleum ether and dried under reduced pressure to give **21** in 67% yield. Yellow solid; yield 67%, mp 248 °C. ^1^H-NMR (CDCl_3_) *δ* 2.17 (3H, s), 2.30 (3H, s), 7.84 (1H, d, *J* = 9.0 Hz), 8.56 (1H, dd, *J* = 9.0 Hz and 2.4 Hz), 8.76 (1H, d, *J* = 2.4 Hz), 9.74 (1H, br s). ^13^C-NMR (CDCl_3_) *δ* 23.2 (CH_3_), 24.3 (CH_3_), 117.0 (C), 121.3 (C), 123.1 (CH), 128.9 (CH), 129.4 (CH), 146.2 (C), 151.7 (C), 152.3 (C), 160.4 (C), (C-NO_2_) was not observed in these experimental conditions. Anal. Calcd for C_12_H_10_ClN_3_O_3_: C, 51.53; H, 3.60; N, 15.02. Found: C, 50.99; H, 3.53; N, 14.94.

## 4. Conclusions

Quinazoline derivatives are molecules with a high pharmaceutical potential, particularly when targeting kinases. Using the consecutive S_RN_1+E_RC_1 radical reaction strategy, a series of original 4-propylaminoquinazolines, bearing a vinylic chloride group at position 2, was prepared in a simple and convenient way from corresponding trichloromethylated substrates. The reaction parameters were studied and optimized by using a phase-transfer procedure involving 5 equiv. of both TBAOH and 2-nitropropane, and the radical mechanism was investigated by employing radical reaction inhibitors such as TEMPO. Finally, the reaction was extended in 4-butylaminoquinazoline, 4-anilinoquinazoline and quinazoline-4(3*H*)-one series, demonstrating that the S_RN_1+E_RC_1 reaction operated onto nitrated quinazolines is compatible with the presence of an alkylamino electrodonating group, so as with a lactam function.
